# Anti-EphA2 Antibodies with Distinct In Vitro Properties Have Equal In Vivo Efficacy in Pancreatic Cancer

**DOI:** 10.1155/2009/951917

**Published:** 2010-01-14

**Authors:** Helenia Ansuini, Annalisa Meola, Zeynep Gunes, Valentina Paradisi, Monica Pezzanera, Stefano Acali, Claudia Santini, Alessandra Luzzago, Federica Mori, Domenico Lazzaro, Gennaro Ciliberto, Alfredo Nicosia, Nicola La Monica, Alessandra Vitelli

**Affiliations:** ^1^Istituto di Ricerca di Biologia Molecolare P. Angeletti, Pomezia, 00040 Roma, Italy; ^2^Instituto de Biología Molecular de Barcelona (IBMB-CSIC), 08028 Barcelona, Spain; ^3^Okairos, Via dei Castelli Romani 22, Pomezia, 00040 Rome, Italy

## Abstract

The EphA2 receptor tyrosine kinase is overexpressed in a variety of human epithelial cancers and is a determinant of malignant cellular behavior in pancreatic adenocarcinoma cells. Moreover, it is expressed in tumor endothelium and its activation promotes angiogenesis. To better clarify the therapeutic potential of monoclonal antibodies (mAbs) directed to the EphA2 receptor, we generated a large number of mAbs by differential screening of phage-Ab libraries by oligonucleotide microarray technology and implemented a strategy for the rapid identification of antibodies with the desired properties. We selected two high-affinity and highly specific EphA2 monoclonal antibodies with different in vitro properties on the human pancreatic tumor cell line MiaPaCa2. One is a potent EphA2-agonistic antibody, IgG25, that promotes receptor endocytosis and subsequent degradation, and the second is a ligand antagonist, IgG28, that blocks the binding to ephrin A1 and is cross-reactive with the mouse EphA2 receptor. We measured the effect of antibody treatment on the growth of MiaPaCa2 cells orthotopically transplanted in nude mice. Both IgG25 and IgG28 had strong antitumor and antimetastatic efficacy. In vivo treatment with IgG25 determined the reduction of the EphA2 protein levels in the tumor and the phosphorylation of FAK on Tyr576 while administration of IgG28 caused a decrease in tumor vascularization as measured by immunohistochemical analysis of CD31 in tumor sections. These data show that in a pancreatic cancer model comparable therapeutic efficacy is obtained either by promoting receptor degradation or by blocking receptor activation.

## 1. Introduction

Eph receptors are a unique family of receptor tyrosine kinases (RTK) that play critical role in embryonic development and in human diseases [1]. The ligands of Eph receptors, called ephrins, are bound to the cell membrane and are involved in cell to cell contact for ligand-receptor interaction. 

Eph-ephrin complexes can generate bidirectional signals that affect both the receptor-expressing and ligand-expressing cells [2, 3]. Eph receptor “forward” signaling depends on the tyrosine kinase domain, which mediates autophosphorylation and phosphorylation of other proteins, and on the association with various effector proteins. Ephrin ligands trigger a “reverse” signaling by association with other proteins. Eph receptor signaling has been implicated in cell-cell repulsion and adhesion, tissue patterning, and angiogenesis [4]. 

EphA2 is over-expressed in different types of cancer including pancreatic, lung, melanoma, colorectal, ovarian, and breast [5, 6]. However, despite the strong correlation of EphA2 receptor expression with malignant phenotypes, the mechanism by which EphA2 contributes to tumor cell malignancy is far from clear [4–6]. Some evidence suggests that EphA2 receptor phosphorylation is not necessary to confer kinase activity and tumorigenicity [7, 8] or is even tumor suppressive [9]. Other data suggest that EphA2 receptor phosphorylation is important in conferring the oncogenic potential [10–12]. Not only tumor cells but also tumor endothelium expresses a high level of EphA2, suggesting a role for the receptor within the tumor cell and in the surrounding tumor microenvironment [10, 13]. 

Targeting of EphA2 with antisense oligonucleotides or monoclonal antibodies (mAbs) inducing proteosomal degradation of membrane-bound receptor reverses breast and pancreatic adenocarcinoma cell growth [14, 15]. Similarly, recent studies showed the effectiveness of soluble EphA2-Fc receptor in inhibiting tumor angiogenesis in a xenograft model of human pancreatic carcinoma [11]. Therefore pancreatic tumor cells appear to be sensitive to EphA2 targeting by different mechanisms. 

The aim of the present work was to generate monoclonal antibodies to evaluate the therapeutic potential of targeting EphA2 in pancreatic tumor. We have developed an antibody that mimics the natural ligand and activates receptor signaling and another which competes with the ligand thereby blocking both “forward” and “reverse” signaling. The data obtained demonstrate that in pancreatic cancer anti-tumor activity can be achieved targeting EphA2 with different mechanisms.

## 2. Materials and Methods

### 2.1. Cell Lines and Clones

Human pancreatic MiaPaCa2, Neuro-2a (N2a), and Hek293 were obtained from American Type Culture Collection and cultured according to specifications. The mouse EphA2-expressing colon carcinoma cell line MC38-CEA has been previously described [16]. To generate the stable Hek293 cell line expressing EphA2 in an inducible manner, 293/EphA2, the human EphA2 cDNA (NM_004431) was cloned from Origene Full Length Clone into the inducible expression vector pCEPTetO-MCS. The resulting vector carried hEphA2 cDNA downstream of CMV promoter. The CMV promoter was preceded by the TetO cassette, allowing EphA2 expression upon doxycycline induction. Stably transfected 293 EBNATet cells clones were selected by hygromycin [17] and EphA2 expression was confirmed by FACS analysis after 16 hours of doxycycline induction. Human full length cDNA clones EphA1, EphA2, and EphA5 (Origene), EphA3, and EphA7 (Invitrogen and GeneCopoeia, resp.) were transferred into pcDNA-DEST40 Vector (Invitrogen) by recombination reaction. Human full length EphA4 cDNA (Open Biosystems) was subcloned into pENTR1A and transferred into pcDNA-DEST40 Vector by recombination reaction. N2a cell line was transfected using Lipofectamine 2000, according to the manufacturer instructions.

### 2.2. Selection of Anti-EphA2 mAbs

Panning of the tagged 4 k Mbr phage-Ab library was performed on induced and not-induced 293/EphA2 as described [18]. 10^7^ cells were incubated with 10^11^ phage. Following extensive washing, cell-bound phage was eluted and used to infect TG1 cells. Ampicillin-resistant bacterial colonies were collected and phagemid DNA purified. Tag sequences were amplified by PCR, labeled with Cy5 or Cy3 dyes, and assembled in Hybridization mix. Images were acquired by Agilent scanner and processed using Feature Extraction software (v 9.1, Agilent Technologies). Rescue of scFv associated to selected tags and scFv conversion to IgG class 1 were performed as described [18].

### 2.3. Apparent Kd Determination of Anti-EphA2 mAbs

MiaPaCa2 cells (3 × 10^5^) were incubated with antibodies or ephrinA1/Fc ligand (R&D Systems) in PBS 1% BSA, 10 mM HEPES (FACS buffer) then binding was revealed by APC-conjugated anti-human Fc antibody (Jackson ImmunoResearch). FACS acquisition and analysis were performed using FACSCanto (Becton Dickinson) and BD FACSDiva software. Mean Fluorescence Intensity (MFI) values were analyzed by Sigma-Plot software. For competition experiments, MiaPaCa2 cells were incubated in FACS buffer with increasing concentrations of the antibodies; then the ligand ephrinA1/Fc labeled with Zenon Alexa Fluor 647 IgG labeling kit (Invitrogen) was added at saturating concentration (30 nM).

### 2.4. Western Blot Analysis of Cell and Tumor Lysates

Approximately 50–100 mgs of frozen tumor were lysed in Mixer Mill MM300 homogenizer (QIAGEN) for 2 minutes in TPER buffer (Pierce) supplemented by 0.5 uM okadaic acid (Calbiochem), protease inhibitor cocktail (Roche), and Halt phosphatase inhibitor cocktail (Pierce). After overnight (ON) starvation, MiaPaCa2 cells were incubated 2 hours in complete medium and 15 minutes with Ctrl IgG, IgG25, or IgG28 (10 *μ*g/mL) and ephrinA1-Fc (5 *μ*g/mL). Cells were lysed in TPER buffer, 10 minutes at 4°C. 30 *μ*g and 50 *μ*g per lane of cell and tumor lysate, respectively, were loaded, transferred onto nitrocellulose, and probed with the following primary antibodies: anti-EphA2, (Santa Cruz Biotechnology), anti-FAK (Upstate), anti-FAKpY^576^ (Biosource), anti-Akt (Cell Signalling Technology cat. 9272), anti-phosphoAkt (Cell Signalling Technology), and anti-phosphoERK (Santa Cruz Biotechnology). Secondary antibodies were antirabbit IgG- HRP diluted (Pierce) or antimouse HRP (Promega). Images were acquired by luminescent image analyzer LAS 3000 and then quantified by MultiGauge ver 2.2 software (FUJIfilm Science). To verify equivalent sample loading, blots were stripped and reprobed for *β*-actin (NeoMarkers).

### 2.5. EphA2 Immunoprecipitation

Cells were incubated with Ctrl IgG, IgG25, or IgG28 (10 *μ*g/mL) and ephrinA1-Fc (5 *μ*g/mL) for 20 minutes at 4°C and for 5 minutes at 37°C. Cell lysates were incubated ON at 4°C with Protein G Sepharose beads (GE Healthcate) coated with anti-EphA2 antibody (Santa Cruz; 0.8 *μ*g/sample). Washed beads were resuspended in SDS loading dye. Recovered proteins were analyzed by western blotting with anti-pTyr 4G10 antibody (Upstate). After stripping the filters were probed with anti-EphA2 antibodies.

### 2.6. EphA2 Internalization Assay

Cells were first precooled at 4°C for 10 minutes in HBSS buffer and then incubated 30 minutes at 4°C with IgG25, IgG28, and ephrinA1-Fc (at 1, 0.2 and 0.5 ug/mL, resp.). Cells were then shifted at 37°C for 0, 30, 60, and 90 minutes, fixed in 2% paraformaldehyde for 15 minutes at RT, and labeled with an antihuman IgG Alexa A647 conjugated (Invitrogen). Secondary antibody was diluted in permeabilization buffer (0.1% Triton;1 mg/mL BSA in PBS). Hoechst was used as nuclear marker. Images were acquired at In Cell Analyzer 1000 instrument. Four fields for each well were acquired at 20× magnification and the internalization was measured by counting the number of positive vesicles per cell by using Multitarget Analysis algorithm.

### 2.7. In Vivo Efficacy Studies

Female athymic nude mice (Harlan) 5 weeks old were maintained in accordance with the guide for the care and use of laboratory animals (IRBM was awarded with Full AAALAC Accreditation since January 2008). 70%–80% confluent MiaPaCa2 cells were harvested, washed, and resuspended in PBS at 2 × 10^7^ cells/mL. Mice were anesthetized under isofluorane inhalation, pancreas was exposed by an abdominal flank incision, and 50 uL of cell suspension were injected subcapsularly in a region of the pancreas just beneath the spleen. Biweekly i.p. treatment with 2 mg/kg of IgG25, IgG28, or Ctrl IgG started 24 hours postcell inoculation. Mice were euthanized at day 35 and tumors were excised, weighed, and either frozen in liquid nitrogen or fixed for immunohistochemistry.

### 2.8. IgG Quantification in Mouse Sera

Mouse sera collected 1 hour, 8 hours, 24 hours, 48 hours, 96 hours, 192 hours, 12 days, 16 days, and 22 days post single IgG administration at 50 *μ*g, were put on ELISA plates coated with antihuman IgG (Bethyl). Purified human IgG was used as standard. Goat antihuman IgG HRP conjugate (Bethyl) was added to reveal the human antibodies. Absorbance at 450 nm was measured with Safire (TECAN).

### 2.9. Immunohistochemistry

Tumors were fixed in 10% buffered formalin and embedded in paraffin. 5 *μ*m microtome sections were cleared in xylol and rehydrated, the unmasking procedure was carried out in antigen retrieve solution (DAKO S1699), 99°C for 40 minutes and then washed and blocked with 1.5% goat serum and 1% Triton in PBS for 20 minutes RT. Anti-CD31 antibody (Pharmingen) was added ON at 4°C and binding was revealed with an antirat antibody (VECTOR labs) incubated for 30 minutes RT. Signal was amplified with ABC kit (VECTOR labs). Sections were counterstained with Herris' haematoxylin and then dehydrated and mounted with Entellan (Merck KGaA). Three sections/samples at three different levels in the tumor were analyzed by AxioVision software (Zeiss). CD31 positive area was measured in each whole section.

### 2.10. Statistical Analysis

Statistical analysis was performed applying the Student's *t*-test. Probability associated with a Student's homoscedastic *t*-test, with a two-tailed distribution, was considered significant when < 0.05. For the analysis of the anti-tumor effect in vivo, we first assessed whether there was a group effect using ANOVA and rejected the null hypothesis of no difference between group and overall means (*P* = .001). We then proceeded with an a priori contrast analysis, assessing if treatments were different from control and different from each other.

## 3. Results

### 3.1. Selection and Characterization of Anti-EphA2 mAbs

We have recently developed a tagArray technology that allows the rapid identification of antibodies out of a library (*Membranome* collection) of phage-displayed antibodies which bind to receptors expressed on the membrane of tumor cell lines [18]. Hek293 cells expressing EphA2 under a doxycycline-inducible promoter (293/EphA2) and the matched noninduced cell line were used as selectors to pan the library. By this approach 30 specific clones were selected and converted into fully human IgG class 1. This strategy generated mainly mAbs which bound to EphA2 conformational epitopes because they did not recognize the denatured form of the protein (data not shown). 

MAb's specificity was confirmed by binding native EphA2 displayed on 293/EphA2 only upon doxycycline induction of receptor expression. The apparent Kd was determined by FACS in a whole-cell binding assay on the human pancreatic cell line MiaPaCa2 and only high-affinity antibodies were selected (Kd < single digit nM). Next, the selectivity of the antibodies was determined after transfection of N2a cells with commercially available cDNAs coding for EphA1, EphA2, EphA3, EphA4, EphA5, and EphA7, followed by whole cell binding assay and FACS analysis. Few antibodies showed some degree of cross-reactivity to EphA4 and were discarded (data not shown). 

**Figure 1:**
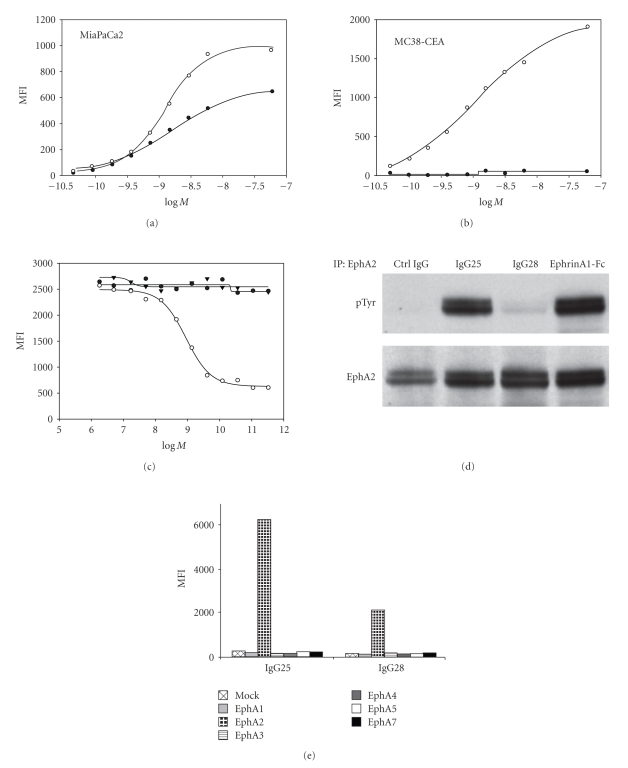
In vitro characterization of IgG25 and IgG28. (a) FACS-based whole cell binding assay was performed with IgG25 and IgG28 on human MiaPaCa2 and (b) on mouse MC38-CEA cells to determine the apparent Kd. Mean Fluorescence Intensities (MFIs) obtained with IgG25 and IgG28 over the logarithm of their molar concentration (LogM) are reported. (c) Binding competition experiments of IgG25 and IgG28 with ephrinA1 on MiaPaCa2 cells, a control isotypic IgG1 antibody (Ctrl IgG) was used as negative control. IgG25 (filled circles), IgG28 (empty circles), Ctrl IgG (filled triangles). (d) EphA2 immunoprecipitation from lysates of cells treated with Ctrl IgG, IgG25, IgG28 and ephA1-Fc, followed by Western Blot with antiphosphotyrosine antibody. After stripping, the same filter was probed with EphA2 antibody as loading control. (e) FACS-based whole cell binding with IgG25 and IgG28 on mouse N2A cells transiently transfected with expression vectors coding for members of Eph A receptor family (EphA1, EphA2, EphA3, EphA4, EphA5, and EphA7).

To identify agonistic mAbs, the level of EphA2 phosphorylation was measured after treatment of MiaPaCa2 cells followed by immunoprecipitation with anti-EphA2 antibodies and Western blot with anti-pTyrosine. Then mAbs were assayed in competition with the ephrinA1-Fc ligand in a whole-cell binding assay. This screening funnel led to the identification of two antibodies, IgG25 and IgG28, whose characteristics are summarized in Figure 1: both antibodies bound to the receptor with high-affinity, IgG25 Kd = 1.3 nM and IgG28 Kd = 1.5 nM (Figure 1(a)). When tested on the mouse EphA2-expressing colon cancer cell line MC38-CEA, only IgG28 showed high affinity cross-reactivity to the murine EphA2 receptor Kd = 1 nM (Figure 1(b)) and was competitive with the ephrinA1-Fc ligand with an IC50 = 0.89 nM (Figure 1(c)). IgG25 triggered receptor phosphorylation in a comparable manner with respect to the ligand, while IgG28 did not induce receptor phosphorylation (Figure 1(d)). Finally, as previously mentioned, both antibodies selectively bound to EphA2 and did not recognize other receptors of the A family (Figure 1(e)).

**Figure 2:**
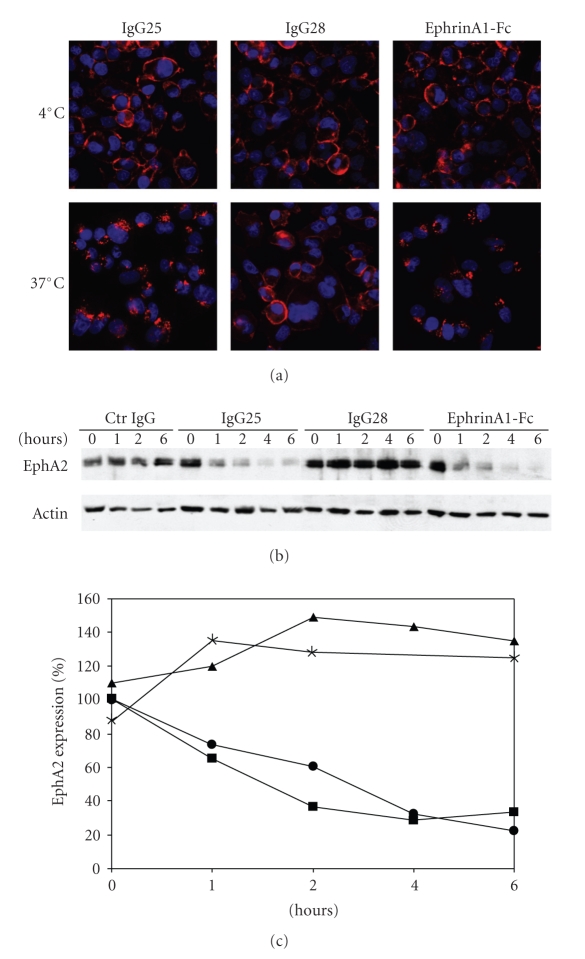
IgG25- and IgG28-mediated EphA2 internalization and degradation in MiaPaCa2 cells. (a) EphA2 internalization in response to IgG25, IgG28, and ephrinA1-Fc treatment. mAbs and ephrinA1-Fc labeled in red while cell nuclei in blue. Images were acquired at 20× magnification. Localization was revealed 1 hour after incubation on cells either at 4°C or at 37°C. (b) Time course Western blot analysis of EphA2 degradation after treatment with control IgG, IgG25, IgG28, and ephrinA1-Fc; anti-*β*-actin was used as loading control. (c) Densitometric analysis of the level of EphA2 expression measured by Western Blot in cells treated with control IgG (asterisks), IgG25 (squares), IgG28 (triangles), and ephrinA1-Fc (circles). Data are expressed as percentage of EphA2 expression over time.

The property of IgG25 and IgG28 to induce receptor internalization and degradation was studied by epifluorescence microscopy and imaging using InCell 1000 and by Western blot analysis of total cell lysates after incubation with MiaPaCa2 cells at various times. Similarly to the ephrinA1 ligand, IgG25 rapidly induced receptor internalization leading to a punctuate staining typical of internalization through the endosomal pathway (Figure 2(a)) and more than 70% of EphA2 was degraded after 4 hours, as shown by Western blot analysis (Figures 2(b) and 2(c)). IgG28 showed a typical membrane staining (Figure 2(a)) and did not trigger receptor degradation (Figures 2(b) and 2(c)).

### 3.2. In Vitro mAb-Dependent Pathway Activation in Pancreatic Tumor Cells

**Figure 3:**
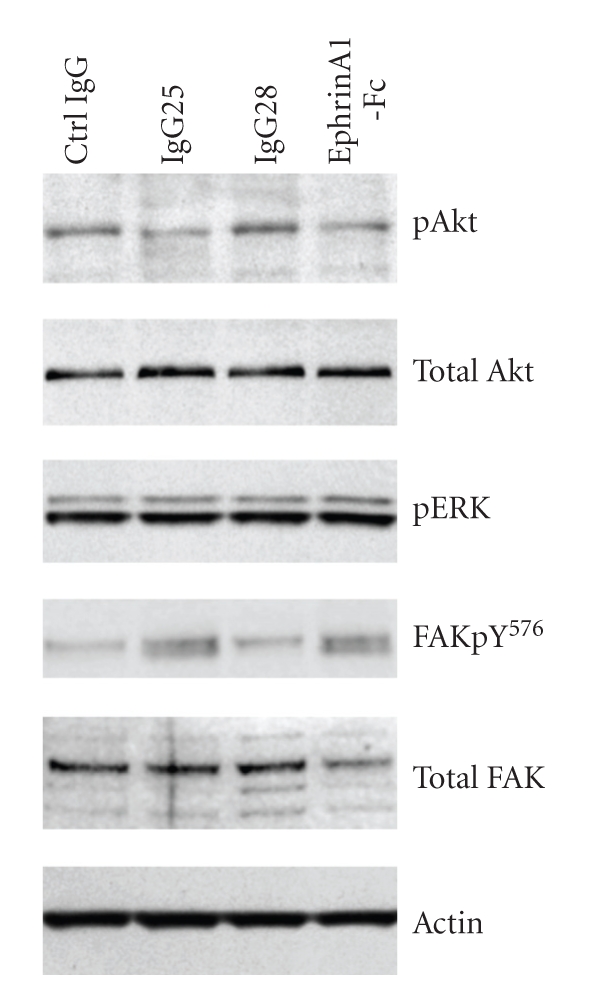
EphA2 downstream signaling in MiaPaCa2 cells treated with IgG25 and IgG28. Cell lysates of MiaPaCa2 were assayed by Western Blotting with antiphospho-Akt, antiphospho-ERK, and anti-phospho FAK (Tyr 576). Antibodies directed to total Akt, total FAK, and actin were used as loading controls.

To investigate EphA2 downstream signaling in MiaPaCa2 cells after incubation with ephrinA1-Fc, IgG25, and IgG28 and with an isotypic IgG control for 15 minutes, total extracts were analyzed by Western blot of pERK and pAkt. As expected, due to the mutant K-RAS harbored by this cell line, both Erk1 and Erk2 were constitutively phosphorylated and signal intensities did not change after treatment with ligand or any of the mAbs (Figure 3). Interestingly, we found a moderate but reproducible decrease in the level of Akt phosphorylation after treatment with both IgG25 and the ligand ephrinA1 while IgG28 treatment did not alter Akt phosphorylation (Figure 3). These data suggest that EphA2 receptor activation in MiaPaCa2 cells leads to downregulation of the PI3Kinase pathway. IgG25 and IgG28 had no effect on MiaPaCa2 cell proliferation as measured by viability assay (ViaLight) or BrdU incorporation (data not shown), though we have shown that in vitro IgG25 and the ligand induced biochemical changes which are known to be proapoptotic. 

It has been reported that EphA2 receptor can interact directly with FAK, an important mediator of growth factor signaling, cell survival, and cell migration. Therefore FAK signaling was studied in MiaPaCa2 cells incubated with IgG25, IgG28, ephrinA1 ligand, and an isotypic IgG control for 15 minutes at 37°C. No association of FAK with EphA2 was detected after immunoprecipitation with antibodies against either of the two molecules (data not shown) and no decrease in FAK phosphorylation was observed. Rather, as shown in Figure 3, when the cells were incubated with IgG25 and ephrinA1 FAK was phosphorylated on Tyr576, IgG28 binding on MiaPaCa2 cells did not induce FAK phosphorylation. These data indicate that EphA2 kinase activation is needed to achieve phosphorylation of FAK on Tyr 576. 

The results of mAb dependent pathway activation confirm that the two EphA2 antibodies have functionally opposite behavior in vitro, IgG25 is an agonist, mimicking the natural ephrinA1 ligand, while IgG28 is an antagonist of EphA2 forward signaling.

### 3.3. Antitumor and Antimetastatic Efficacy of mAbs in MiaPaCa2 Orthotopic Tumor Xenograft Model

**Figure 4:**
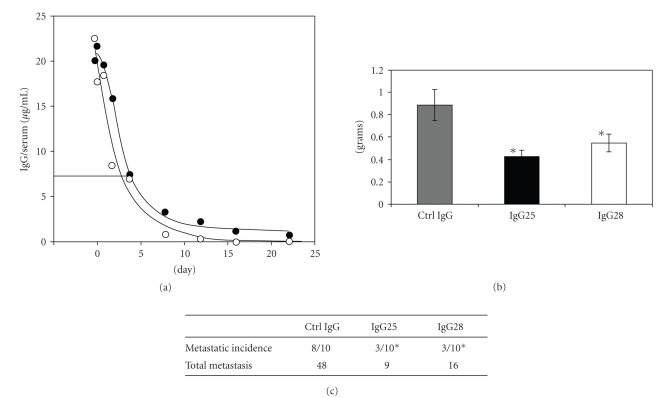
In vivo activity of IgG25 and IgG28 in MiaPaCa2 orthotopic xenografts. (a) Pharmacokinetic analysis of IgG25 (empty circle) and IgG28 (full circle) following a single dose at 2 mg/kg. The straight line represents the trough level of the antibody before the next administration. (b) Tumor weight at day 35 following biweekly administration of control IgG, IgG25, and IgG28. Data, expressed in grams, represent the average of tumor weight from ten different animals (*N* = 10). Error bars indicate the standard error. (c) Impact of IgG treatment on metastatization: the metastatic incidence is given by the number of mice with metastases in each group. The total number of metastases counted for each group is also reported. Metastatic lesions were normally distributed in liver, spleen, stomach, diaphragm, and, in few cases, kidney tissues. Involvement of local lymph nodes was not detected. Asterisks indicate statistically significant differences with respect to the control group (Student's *t*-test; *P*<.05)

The in vivo anti-tumor property of each antibody and the effect of their combination have been studied in a mouse xenograft model of human pancreatic cancer. Ten mice orthotopically implanted with 1 million MiaPaCa2 cells into the pancreas were treated biweekly with 2 mg/kg of IgG25 and IgG28 alone or in combination. A control group was treated with the same dose of an isotypic human IgG1. Dosing schedule was established on the basis of preliminary mAb pharmacokinetic analyses in mice. Terminal half life of 144 and 72 hours was measured for IgG25 and IgG28, respectively (Figure 4(a)). This difference could be explained by a “metabolic sink” due to IgG28 binding to the mouse receptor. Repeating the dose every 72 hours, the trough level of both antibodies remained above the concentration needed for receptor saturation, as confirmed by FACS analysis of MiaPaCa2 cells incubated with serum withdrawn 72 hours after mAb administration (data not shown). Thirty-five days after implantation mice were euthanized, tumors weighted, and metastases counted after necropsy. Data shown in Figures 4(b) and 4(c) demonstrate that both IgG25 and IgG28 induced a strong reduction (50% on average) in primary tumor weight and in the number of metastases. These data were statistically significant either when applying a *t*-test analysis (IgG25 *P* = .002; IgG28 *P* = .018), or when applying a more complex analysis using ANOVA (see Material and Methods) from which we concluded that treatment groups were indeed different from the control group (*P* = .0004) but no difference was observed between the two IgG treatments (*P* = .28). 

Notably, administration of IgG28 which is cross-reactive to the mouse EphA2 receptor did not cause weight loss or clinical signs in any of the treated animals for the duration of the study. Combination of IgG25 and IgG28 did not result in additive inhibition of tumor growth or in the number of metastases (data not shown). 

**Figure 5:**
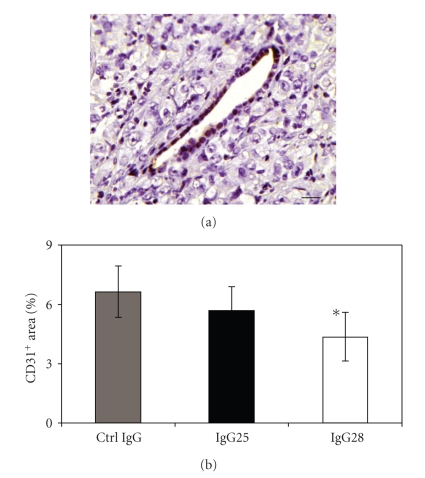
Effect of EphA2 antibodies on angiogenesis. (a) Representative CD31 immunostaining of paraffin embedded tumor section (magnification bar = 20 m). (b) Quantitative analysis of CD31 staining in tumors treated with control IgG, IgG25, and IgG28. The average data obtained from the analyses of two tumors selected from each group are reported as the percentage of CD31^+^ area in each entire section. In all panels, asterisks indicate statistically significant differences with respect to the control group (Student's *t*-test; *P*<.05).

In light of the role of EphA2 in angiogenesis [11, 19, 20], the impact of IgG25 and IgG28 administration on tumor vascularization was also monitored. Quantitative immunohistochemical (IHC) analyses of paraffine-embedded tumor sections were performed to evaluate the level of vasculature in tumors from control and treated mice by CD31 immunostaining (Figure 5(a)). The results showed a moderate but statistically significant decrease in the percentage of CD31 positive staining in tumors treated with IgG28 with respect to untreated controls (*P* = .04) (Figure 5(b)). 

These data show for the first time that a selective antagonist of EphA2 signal transduction has therapeutic efficacy in pancreatic cancer.

### 3.4. EphA2 Expression in Tumors

When explanted, 35 days after continuous mAb administration, tumor masses were still growth inhibited as revealed by the lower weight of treated tumors versus controls. To gain information on the mechanisms underlying the anti-tumor property of the two antibodies, studies on EphA2 downregulation and on activation of EphA2-related signaling molecules were performed on tumor samples from control and treated mice. Total level of EphA2 expression in tumor lysates was evaluated by Western blot analysis. 

**Figure 6:**
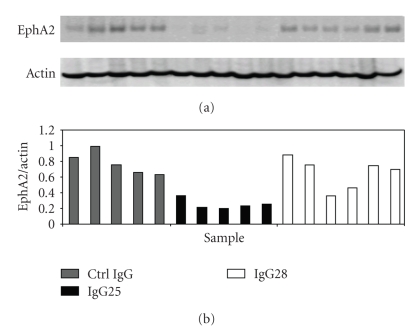
EphA2 protein expression in tumors from treated mice. (a) Western Blot analysis with anti-EphA2 antibodies of  lysates from five tumors treated with control IgG, with IgG25, or six tumors treated with IgG28. (b) Densitometric analysis of the ratio between EphA2 levels revealed by anti-EphA2 antibody in nonsaturating conditions and actin expression measured with antiactin antibody. Data on *y* axis are expressed as arbitrary units.

As shown in Figure 6, IgG25 administration caused a strong decrease of EphA2 receptor level (70% on average) compared to untreated tumors (*P* = .0005), in line with what previously reported on EphA2 agonistic antibodies. On the contrary, tumors treated with IgG28 showed a certain degree of variability in EphA2 receptor levels but no statistically significant difference from the untreated controls (*P* = .22). 

To rule out the possibility that the decreased level of expression in IgG25-treated tumors might be due to altered transcriptional level or to an EphA2 negative cell population arising from the selective pressure of mAb treatment, EphA2 mRNA was quantified in tumor lysates by qPCR, but no variation in the amount of transcript was detected between the different groups (data not shown).

### 3.5. Effect of mAb's Treatment on EphA2 Signaling In Vivo

**Figure 7:**
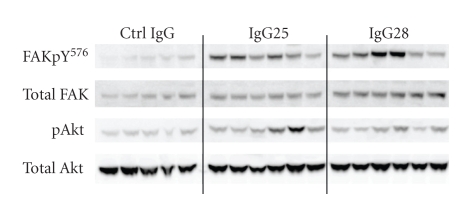
IgG25 and IgG28 modulate in vivo EphA2 downstream signaling: Western Blot analyses of tumor lysates with antiphospho FAK (Tyr 576) and anti-phospho Akt. Antibodies to total Akt, total FAK, and actin were used as loading controls. *P* values were calculated with respect to average densitometric value of group treated with control IgG.

To determine the impact of antibody treatment on the pathways downstream the EphA2 receptor in vivo, we determined the level of expression and phosphorylation of Akt, Erk, and FAK in tumor lysates from control and treated mice. No change was observed in the levels of both total and pErk (data not shown), which was constitutively activated in vivo as it was previously observed in vitro. Differently from what observed in cultured cells, Akt was only poorly activated in untreated tumors but the level of phosphorylation increased about 2-fold on average in the case of tumors from mice treated with IgG25 (*P* = .005) and more modestly 1.6-fold although statistically significant (*P* = .04) in tumors treated with IgG28 (Figure 7). Finally, both IgG25 and IgG28 were able to induce an average of 3-fold increase in FAK phosphorylation on Tyr576 (resp. *P* = .005 and *P* = .013) (Figure 7). Also this latter observation differs from what was observed in cultured MiaPaCa2 cells where IgG28 treatment did not induce FAK phosphorylation (Figure 3).

## 4. Discussion

To study the role of the EphA2 receptor in a pancreatic cancer model two monoclonal antibodies with different functional properties have been selected and characterized. 

One of the novel findings of the present study is the identification of a high-affinity monoclonal antibody, IgG28, selective for EphA2 and cross-reactive with the mouse receptor which blocks the binding of the ephrinA1 ligand to EphA2 expressed on human epithelial cells and on mouse endothelia. When administered to mice orthotopically transplanted with pancreatic MiaPaCa2 tumor cells, IgG28 inhibited tumor progression and metastasis formation and caused decreased tumor angiogenesis (Figures 4 and 5). Moreover, blocking the mouse EphA2 receptor with continuous treatment of IgG28 was not associated to overt toxicity for the duration of the study. The observed inhibitory effect on tumor vasculature was in agreement with previous studies where a soluble EphA2/Fc receptor was used as a receptor antagonist to block endogenous EphA2 signaling [11, 19, 20]. Differently from the highly selective IgG28 antibody, the soluble EphA2 receptor can promiscuously interact with different ephrin ligands and block multiple EphA receptor signaling pathways in the tumor endothelium. Therefore, the precise target of such therapeutic approach remains undefined. By contrast, the use of a selective antagonist allows focusing on the role of the EphA2 receptor in tumor progression in vivo. 

The efficacy of the antagonistic antibody IgG28 was compared with that of an agonistic antibody, IgG25, which mimics the ephrinA1-Fc ligand in potency and kinetics of EphA2 activation and degradation (Figure 2). The anti-tumor efficacy of the administration of either of the two antibodies in xenograft models of MiaPaCa2 tumors was comparable (Figure 4). Contradictory results have been reported on the effects of antibodies that decrease EphA2 expression in several epithelial tumors. A strong anti-tumor activity has been reported using agonistic antibodies that induce receptor phosphorylation followed by internalization and degradation [14, 20]. In contrast, agonistic antibodies have been shown not to inhibit the growth of breast and colon tumor xenograft despite strong downmodulation of EphA2 in tumors [21]. Here, we confirm that IgG25 which triggers receptor activation and downregulation was able to inhibit the growth of a pancreatic xenograft model. 

EphA2 agonistic antibodies are thought to function by restoring a signal that is normally provided by receptor-ligand binding but is lost in most cancer cells due to poor receptor-ligand interactions [22]. To characterize the response of cultured MiaPaCa2 cells to the antibody treatment, a number of in vitro studies have been performed. These cells harbor a mutated K-Ras and show constitutive activation of pErk which does not change after exposure to ephrinA1 or to the agonistic mAb, IgG25. A recent study has demonstrated that EphA2 is a direct transcriptional target of the Ras-Raf-MAPK pathway and that EphA2 signaling contributes to a negative feedback loop regulating Ras activity in a ligand dependent manner [23]. On the contrary, another recent study in pancreatic cells lines showed that EphA2 activation weakly stimulates Erk phosphorylation in MiaPaCa2 cells [24]. The explanation for these differences is not obvious, although it should be noted that different conditions of cell culture, serum starvation, and duration of ligand incubation could contribute to the variability of the results. 

Both ephrinA1 and IgG25 decrease pAkt levels (Figure 3). This observation is in contrast with previous data on MiaPaCa2 cells where Chang et al. found increased Akt phosphorylation upon EphA2 activation [24] but is consistent with a recently published model in which the EphA2 receptor mediates negative feedback inhibition of PI3K-Akt in the presence of constitutive activation of the Ras-Raf-MAPK pathway [25]. Furthermore, ephrinA1 and IgG25 induce FAK phosphorylation on Tyr 576 (Figure 3), confirming what already observed in NIH3T3 cells and in PC3 prostatic cancer cells [26, 27]. It is important to note, however, that the often contrasting data reported on Erk, Akt, and FAK signaling by EphA2 are context dependent and no coherent picture has come up to date on the role of such complex network in Eph-dependent tumor progression [1]. 

When we compared the data relative to EphA2 signaling obtained from cultured MiaPaCa2 cells treated with the antibodies with those obtained in MiaPaCa2 tumors explanted from animals after prolonged antibody administration we observed several discrepancies. Differently from what observed in vitro, tumors treated with IgG28 showed FAK Tyr 576 phosphorylation. Akt, which in cultured cells showed a basal level of constitutive activity, was instead not phosphorylated in untreated tumors but became activated upon antibody treatment. One might speculate that the biochemical signature observed after prolonged treatment with the antibodies is the result of cellular adaptation. Therefore, ongoing studies are focused on the investigation of the early response of the tumor cells to the antibody treatment. 

The data indicate that the most striking correlate to the anti-tumor efficacy of IgG25 is receptor downregulation. This suggests that the mechanism by which reduction of EphA2 expression is achieved is probably not relevant for the growth inhibition of pancreatic tumors in mice. In fact a significant impact on tumor growth has also been reported by siRNA-mediated knock down of EphA2 expression [15]. In contrast, the most likely correlate for the antitumor effect of IgG28 is the reduced angiogenesis in the tumor. The lack of any additive therapeutic effect in the coadministration of the two antibodies may be due to the counteracting activity on tumor receptor downregulation which results in neutralization of growth inhibition. 

While the present work demonstrates the usefulness of monoclonal antibodies as tools for dissecting the therapeutic potential of membrane receptor targets in tumorigenesis, it also stresses the need for a thorough in vivo investigation of antibody-based therapeutics to identify the real pharmacodynamic marker that correlates with anti-tumor efficacy.
